# Knowledge, perception about antiretroviral therapy (ART) and prevention of mother-to-child-transmission (PMTCT) and adherence to ART among HIV positive women in the Ashanti Region, Ghana: a cross-sectional study

**DOI:** 10.1186/1472-6874-13-2

**Published:** 2013-01-22

**Authors:** Daniel Boateng, Golda Dokuaa Kwapong, Peter Agyei-Baffour

**Affiliations:** 1 Department of community Health, Kwame Nkrumah University of Science and Technology, Kumasi, Ghana; 2 The United States Agency for International Development (USAID)/ Focus Region Health Projects, Accra, Ghana

**Keywords:** ART, PMTCT, HIV, Women, Ghana

## Abstract

**Background:**

Mother-to-Child Transmission (MTCT) has been identified as the greatest means of HIV infection among children. Adherence to antiretroviral drugs is necessary to prevent drug resistance and MTCT of HIV among HIV positive women. However, there is a gap in clients’ knowledge, attitudes and perceptions of antiretroviral therapy (ART) and Prevention of Mother-To-Child Transmission (PMTCT) which influence their decision to adhere to ART.

**Methods:**

The study was a descriptive cross-sectional employing both qualitative and quantitative methods. The study involved 229 HIV positive women in reproductive age (18 – 49 years) and had been on ART for at least six months. Fourteen health workers were also included in the qualitative study. Respondents were selected from three ART centers in the Kumasi Metropolis through systematic random sampling from August to November 2011. HIV positive women who had consistently missed two or more ART appointments within the previous two months were classified as defaulters. Data was analyzed with SPSS 19 and STATA 11. Logistic regression was run to assess the odds ratios at 95% confidence level.

**Results:**

The ART defaulter rate was 27% and clients had good knowledge about ART and PMTCT. More than 90% of the HIV positive women had inadequate knowledge about ART and PMTCT and these women were more likely to default ART (OR = 3.5; 95% CI = 1.89, 6.21). The educational background of HIV positive women did not have significant influence on their knowledge of ART and PMTCT.

**Conclusions:**

Mothers, knowledge and understanding of ART and PMTCT could influence their adherence to ART. Educational interventions which target the understanding of both the literate and illiterate women in society are necessary to develop positive behaviors and enhance adherence to ART.

## Background

HIV remains the leading cause of death in Sub-Saharan African [1] and HIV infection among children has mainly been through Mother-To-Child-Transmission (MTCT) [2]. However, the most effective way of preventing MTCT of HIV is to prevent infection in women of reproductive age. Nearly 16 million women are living with HIV with 1.4 million pregnant women at risk of passing along HIV to their children each year [3]. In Sub-Saharan Africa, the continent most ravaged by the epidemic, females are close to 60% of those infected with the virus and form 75% of infected 15–24 year olds. In Ghana, the level of HIV infection in 2009 was nearly 3 times higher among young women (1.3%) than young men (0.5%) and in 2010, new infections of HIV among females were higher than males (7,039 vs 5,852) [4,5]. This growing feminization of the HIV pandemic does not only reflect women’s greater physiological vulnerability to infection, but also their social and psychological vulnerability created by a set of interrelated economic, socio-cultural and legal factors [6].

In Ghana, Prevention of mother-to-child transmission (PMTCT) services are based on the United Nations adopted recommendations from the Inter-Agency Task Team (IATT) on prevention of MTCT of HIV for the implementation of a comprehensive four-pronged strategic approach [7]. This comprises; primary prevention of HIV among women of reproductive age; prevention of unintended pregnancies among women living with HIV; prevention of HIV transmission from women living with HIV to their babies; and provision of appropriate treatment, care and support to mothers living with HIV and their families [7]. Though awareness of HIV and AIDS have been high since 2003, where 98% of women and 99% of men were reportedly aware of HIV, comprehensive knowledge on HIV and AIDS, appropriate prevention and non-stigmatizing behaviour have been lagging behind [8]. In 2008, the Ghana Demographic and Health Survey (GDHS) showed that only 28.3% of female respondents age 15–24 and 34.2% of men had comprehensive knowledge about HIV and AIDS. There has thus been little progress along this front [9].

Patients’ knowledge and practices on HIV/AIDS, PMTCT and ARTs have been shown to influence their motivation and uptake of ARVs for PMTCT [10,11]. As reported by Wenger et al. [12], a good level of understanding about HIV by the patient, a belief that ART is effective and prolongs life and recognition that poor adherence may result in viral resistance and treatment failure, could impact favorably upon his/her ability to adhere. Conversely, lack of interest in becoming knowledgeable about HIV and a belief that ART may in fact cause harm adversely affect adherence [12]. Previous studies on the continent have found mothers knowledge on PMTCT to be low [13,14].

Knowledge of HIV, ART and PMTCT could however be influenced by interplay of socio-economic and other cultural factors including clients’ educational level [11]. A higher level of education has a positive impact on patient’s ability to adhere to ART [11]. This paper sought to (i) assess the level of knowledge and perceptions of clients on ART and PMTCT and (ii) determine the extent of influence of clients’ knowledge level on accessing ART.

## Methods

### Study setting

A descriptive cross sectional study involving the use of both qualitative and quantitative data collection techniques was employed. The study was conducted in Kumasi, the capital city of the Ashanti region and the second largest city in Ghana. The metropolis is bounded in the north by Kwabre, Bosomtwe and Atwima Kwanwoma to the south; on the east is Ejisu and Atwima is on the west of the metropolis. The Kumasi metropolis is the largest of the twenty-seven (27) political divisions (metropolis, municipality, districts) in Ashanti Region. It has an estimated population of 1,430,241 with an annual growth rate of 3.4%. The population is distributed in about seventy-six (76) communities in the metropolis. The Ashanti Region has 152 PMTCT centres and 21 ART centers. There are eight ART centres in the Kumasi Metropolis. As of 2010, the Ashanti Region was second with 3.9% to Eastern Region (4.2%) in the prevalence of HIV in the country with the prevalence being higher in the urban cities than the rural communities [15].

### Study population and sample

The study was conducted in ART centres across Kumasi. The study population consisted of consenting HIV positive women who had been put on ARVs for treatment or prophylaxis (receiving ARVs for PMTCT) for at least six months, aged 18 to 49 years. A total of 206 women were randomly selected for the study from the ART centres at the various facilities for the quantitative study. The sample was selected in two (2) stages. Three facilities were selected out of a total of eight (8) ART centres in the Metropolis. All the names of the eight centres were numbered and put in a bowl and three; Suntreso Government Centre, Aninwaa Medical Centre and Kumasi South Government Hospital were selected without replacement. Systematic random sampling technique using the average weekly attendance was used to select respondents through exit interviews. Administrative records, which included the pharmacy refill, register and medical consultation appointment visits were also reviewed.

In the qualitative study, a total of 23 HIV positive women and 14 health workers were involved. These were included in three Focus Group Discussions (FGDs) with 8 participants each at Suntreso and Kumasi South Hospitals and 7 participants at the Aninwaa Hospital. The health workers participated in in-depth interviews.

### Data collection and statistical analysis

#### Quantitative study

The quantitative data collection was done with structured questionnaire. Questionnaires and interview guides were pre-tested to check for clarity, consistency and acceptability of the questions to respondents. Following this, the necessary corrections were made and questionnaires finalized for the actual field work. All questionnaires and interview results from the field were checked for completeness and internal errors during data collection. Questionnaires were then sorted, numbered and coded before entering using SPSS software.

Data was analyzed using SPSS 19 and STATA 11. The main outcome of the study was respondents’ knowledge level on ART and PMTCT. Responses on the various questions to test for knowledge were coded as “yes”, “no” or “don’t know”. HIV positive women who had consistently missed two or more ART appointments within the previous two months were classified as defaulters. General knowledge level was computed by respondents’ total correct responses from the various issues posed to test for knowledge. Respondents who accepted all correct responses were grouped having “adequate knowledge” and vice versa. Bivariate associations and 95% confidence intervals were used to access the influence of selected socio demographic characteristics on the knowledge level of the women. The demographic factors included age, marital status (single and married), religion and educational level. The educational level was classified into formal (respondents who had ever completed a structural and certified school program). Single marital status involved women who had never married, divorced or widowed. The median time on ART for study population was 15 months.

#### Qualitative study

Data was obtained through FGDs and in-depth interviews with key informants using tape recorders and interview guides. Interviews and the FGDs were carried out in quiet and discreet locations in the hospital’s outpatient department and were conducted and audio-taped in the local language. Tapes were transcribed verbatim in Twi and then back-translated into English. Spot checks of interview and FGD transcripts and translations were regularly conducted to ensure the completeness of the transcription and the accuracy of the translation. Qualitative data was analyzed using Atlas.ti. A preliminary analysis of interviews was done and used for validation of results and further exploration using focus group interviews.

### Ethical consideration

Ethical clearance for the study was obtained from the Committee on Human Research, Publications and Ethics (CHPRE), the institutional Review Board of the Kwame Nkrumah University of Science and Technology (KNUST) and Komfo Anokye Teaching Hospital (KATH). Participants were given consent form to sign and had all their concerns and questions answered before data collection began. Anonymity and confidentially of the participants was assured since there was no inclusion of any identifiers or incriminating information on participants. Translators were used for participants who could not read the English language.

## Results

### Socio demographic characteristics

Respondents’ ages ranged between18 and 49 years with a mean age of 35 (standard deviation =7.2). Majority of the respondents (87%) were Christians and more than half (55%) were married. Eighty-seven percent of the husbands had formal education and 84% were employed. Majority of the respondents had completed Junior Secondary school while 21% had no formal education. More than two thirds were employed (70%) and earned less than GHS 200.00 ($105.46) monthly.

### Knowledge on PMTCT and ART

The analysis of data indicated that, in most items respondents had good knowledge about HIV/AIDS, PMTCT and ARVs. The percentages of 'true' responses for most of the knowledge items were higher than 'false' and 'don't know'. Respondents’ knowledge on the mode of transmission of HIV was very high. Almost all the respondents knew that transmission was through sexual intercourse (98.5%) and the use of unsterilized instrument (96.6%). The rest included blood transfusion, (91.3%) and 87.7% believed transmission could be through mother to child transmission (vertical). Fifteen percent of respondents still held the view that transmission could be through spiritual means, Table 1.

**Table 1:** Knowledge about HIV/AIDS, PMTCT and ART

**Variable**	**Total**	**N**	**%**
Mode of spread of HIV/AIDS*			
Sexual intercourse	206	203	98.5
Blood transfusion	206	188	91.3
Unsterile instrument	206	199	96.6
MTCT	206	179	87.7
Other (spiritual)	206	33	15.0
**Mother to child transmission**			
Possible	206	190	92.2
Not possible	206	1	0.5
Don’t know	206	15	7.3
Mode of mother –to-child transmission*			
In the womb (intrauterine)	190	179	94.2
During delivery	190	154	81.1
Through breastfeeding	190	187	98.4
**MTCT preventable?**			
Preventable	206	182	88.3
Not preventable	206	8	3.8
Don’t know	206	15	7.2
Means of PMTCT*			
Giving ART	182	176	96.7
Avoiding breastfeeding	182	162	89.0
C/S delivery	182	69	37.9
Antiretrovials*			
Drug to prevent transmission to baby	206	58	28.2
Drug to cure HIV	206	78	37.9
Drug to prevent death from HIV/AIDS	206	147	71.4
Drug to prolong lives	206	184	89.3
Works effectively with optimal adherence	206	200	97.6

*Multiple responses.

Majority of the respondents (92%) perceived that mother-to-child transmission of HIV/AIDS was possible, whereas (1%) did not consider that as a possibility. Few (7.3%) did not know if MTCT of HIV was possible. As detailed in Table 1, vast majority of those who perceived that mother-to-child transmission is possible, had good knowledge about the means of MTCT. More than 90% knew MTCT could be intra-uterine, 81.1% during delivery and 98.4% through breastfeeding. Knowledge on PMTCT and ART was also good among respondents. As high as 88% knew that vertical transmission was preventable whiles 7.2% did not know that MTCT was preventable. On ART, 62% knew that ART is not given to cure HIV/AIDS and almost all the respondents (97.6%) knew that ARVs can be effective only on optimal adherence.

In the qualitative study, some respondents still had negative perceptions about the causes of HIV/AIDS. Few thought HIV is by bewitchment and they have been seeking spiritual intervention at prayer camps and spiritualists.

*“I believe the only means I might have gotten the disease was spiritual because I brought my husband and children to test after I was tested positive and none of them had got it. I have not committed adultery and I don’t go to the hairdresser's salon. I believe someone bought it for me spiritually.”* (ARV user, Kumasi South Government Hospital).

Most of the respondents had good knowledge on PMTCT and ARVs. Most of the women stated that, the drugs makes the virus weak and unable to attack their immune system.

*“The drug acts like a cup to cover the virus and prevents them from acting so I only need to take it always to keep them where they are, and I can live as long as God wants me to”* (ARV user, Suntreso Government Hospital).

*“When I tested positive, I was referred to this place to take ARVs. I know I need the drugs to prevent my baby from getting infected and this is very important to me so I make sure I come whenever I'm supposed to” (*ARV user, Aninwaa Hospital).

The health workers highlighted on the institution of adherence counseling to counsel clients on the benefit of adherence and the dangers of defaulting ART. They disclosed how this has improved adherence.

*“All clients go through adherence counseling before initiating ARVs. When they default along the way, they begin counseling all over for three weeks and this has been discouraging defaulting because they don’t want to go through that so they always make sure they come when it is time.”* (Prescriber, Kumasi South Hospital).

### Influence of knowledge on PMTCT and ART on default of ART

**Figure 1:**
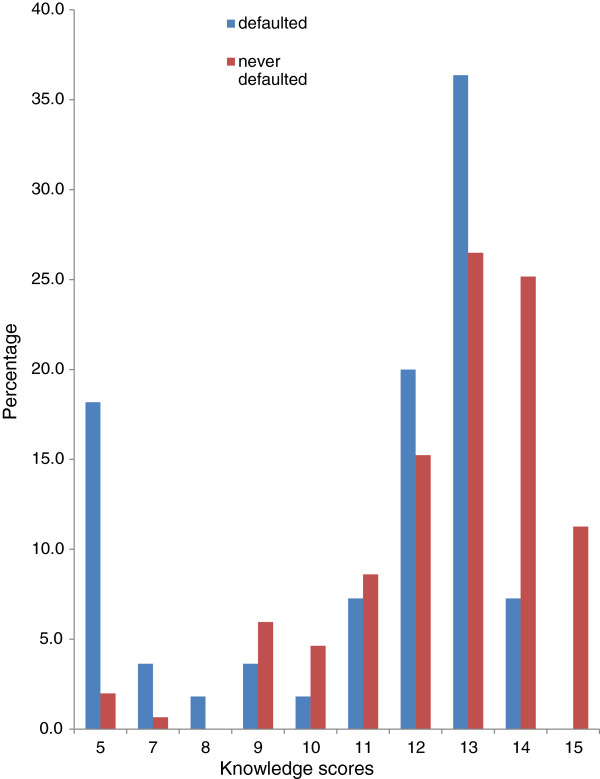
Knowledge scores of ART and PMTCT among defaulters and non-defaulters of ART.

As shown in Figure 1, only 17 respondents provided correct responses to all questions to tease out level of knowledge of ART and PMTCT. This constituted only 11% of those who had never defaulted ART and 8% of total respondents. Defaulting rate was significantly higher among HIV positive women with inadequate knowledge on PMTCT and ART as compared to those with adequate knowledge (29% versus 0%), Table 2. Women with inadequate knowledge were 3.5 times more likely to default ART as compared to those with adequate knowledge and this difference was statistically significant (OR = 3.5; p < 0.01), Table 2.

**Table 2:** Relationship between knowledge level and access to ART

**Knowledge level**	**Defaulted**	**Never defaulted**	**Total**	**OR (95% CI)**
	**N (%)**	**N (%)**	**N (%)**	
**Adequate**	0 (0)	17 (100)	17 (8)	1
**Inadequate**	55(29)	134 (71)	189 (92)	3.5 (1.89, 6.21)**
**Total**	55 (27)	151 (73)	206 (100)	

### Influence of socio-demographic characteristics on knowledge level of PMTCT and ART

In a comparative analysis (Table 3), adequate knowledge was higher among clients aged beyond 44 years (10%) and clients who were married had higher level of adequate knowledge as compared to those who were not married (11% vrs 5%) although this was not statistically significant. Percentage of HIV positive women with adequate knowledge were 9% among those with formal education and 6% among those with no formal education. Adequate knowledge was again higher among the Christians than the Muslims although this difference did not reach significant levels (10% vrs 0%).

**Table 3:** Extent of influence of socio demographic characteristics on knowledge level

**Co-variates**	**Knowledge level**		**Odds ratio**	**95% CI**
	**Adequate**	**Inadequate**		
**Age**				
<25	1 (8)	11 (92)	1	
25-34	7 (9)	75 (91)	0.97	0.11, 8.72
35-44	6 (7)	77 (93)	0.81	0.09, 7.46
>44	3 (10)	26 (90)	1.28	0.12, 13.91
**Marital status**				
Single*	5 (5)	88 (95)	1	
Married	12 (11)	101 (89)	1.63	0.44, 5.95
**Education**				
No formal	3 (6)	49 (94)	1	
Formal	14 (9)	139 (91)	1.74	0.47, 6.42
**Religion**				
Christian	17 (10)	160 (90)	1	
Muslim	0 (0)	27 (100)	0.42	0.13, 1.30

*Single = divorced, widowed, never married.

**Figure 2:**
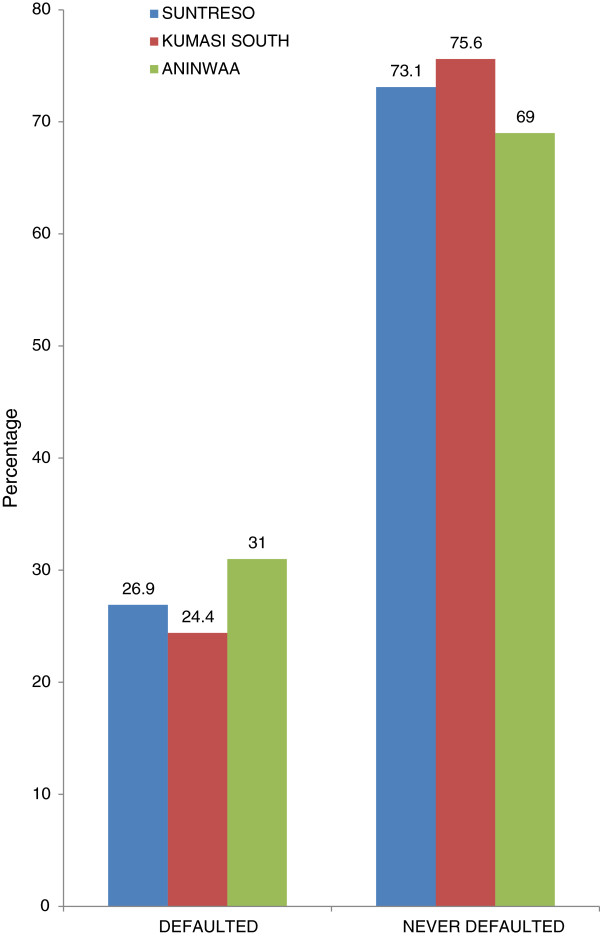
ART defaulting rate by facility.

Fifty-five respondents representing 27% had missed their ART appointment within the previous two months whiles 73% had never missed an ART appointment. The defaulter rate among respondents was 26.9% in Suntreso Government Hospital, 24.4% in Kumasi South Government Hospital and 31.0% in Aninwaa, Figure 2.

## Discussion

Earlier studies postulated a positive relationship between knowledge level and utilization of services [12-14]. However, there is little evidence of the influence of the knowledge and perceptions of ART and PMTCT of HIV positive women on their utilization of PMTCT services in Kumasi Metropolis, which is needed to inform the design of new intervention and uptake of existing ones. We explored the knowledge and perception and their predisposing factors through interviews with 229 women who were HIV positives. The general defaulting rate was 27% with variations in the rates at the various facilities. This was much lower and inconsistent with estimates of average rates of adherence to ART in many different social and cultural settings which range from 50% to 70% [16-20].

Generally, respondents had good knowledge on ART and PMTCT and this could be partly due to the institution of counseling as part of the programme, where new clients are taken through the benefits of adhering to ART, the problems associated with defaulting ART and issues relating to PMTCT. The respondents who had achieved optimal access cited the need to adhere to treatment to ensure effectiveness of the drug as a reason for always coming for ART appointment. This to some extent implies that the counseling had been very beneficial to the patients. Only 8% of respondents however had adequate knowledge on ART and PMTCT. Patient’s knowledge and practices on HIV/AIDS, PMTCT and ARTs influenced their motivation and uptake of antiretrovirals for PMTCT. HIV positive clients with inadequate knowledge in this study were significantly more likely to miss ART appointments as compared to those with adequate knowledge. As indicated in the study by Wenger et al. [12], HIV patients are less likely to miss ART appointments when they have a good knowledge of the etiology of the disease and understand that ART is effective upon adherence. This is also supported by studies by Catz et al. [11] and Duff et al. [10], where knowledge level was statistically significantly associated with access to ART (p < 0.001).

The result was in line with a report by Dzokoto [8], which indicated that knowledge on HIV and AIDS has been increasing since 2003, where 98% of women and 99% of men were reportedly aware of HIV. The result was also consistent with a recent study [14], where knowledge on PMTCT was high among the women studied. In that study, majority of the mothers knew that it was possible to reduce the risk of transmission during pregnancy (82.2%) and the breastfeeding period (71.6%). Eighty-eight percent knew vertical transmission is preventable and 85% knew it can be done through giving ART. However, only 34% were aware that delivering through cesarean section (C/S) has the possibility of reducing vertical transmission of HIV/AIDS.

Breastfeeding approximately doubles the risk of mother-to-child transmission and HIV positive mothers’ knowledge of breastfeeding as a means of MTCT is necessary to inform their decision to breastfeed or not. In populations where breastfeeding continues into the second year, the risk of transmission through breastfeeding increases by about an additional 15-20%, in absolute terms. The risk of transmission during breastfeeding is more or less constant and continues as long as breastfeeding continues, at a rate of approximately 1% per month of breastfeeding [21].

The knowledge on C/S as a means of preventing MTCT was low among the women. Other factors exerting an independent effect on MTCT include pre-term delivery, rupture of membranes (every hour of membrane rupture increases the risk of infection by 4%) [22]. HIV positive women must be aware of C-section as means of PMTCT although it could not be recommended as an option in resource limited settings. Elective caesarean delivery before labor and rupture of the membranes reduces the transmission risk by approximately half [22,23].

A lower level of general education and poorer literacy may impact negatively on some patients’ ability to adhere, and vice versa. Women with formal education were adequately knowledgeable about ART and PMTCT as compared to those without formal education. This could impact positively on their ability to adhere since they understand the ART, PMTCT and the need for them to adhere to ensure the effectiveness of the drug and also to prevent transmission to their babies when pregnant. This could be due to the fact that current educational packages are more understood and acceptable to the literate and they are also more able to read and understand educational materials. Most sensitization media including bill boards, TV adverts and leaflets as part of the social marketing campaign strategies are conducted in English language making it difficult for the illiterate in society to understand. This demands redesigning of educational interventions that targets both the literate and illiterate women.

Some respondents still held the opinion that HIV is a spiritual disease and therefore there is the need to seek spiritual interventions. Some defaulters cited “use of alternative medicines “as a reason for not going for antiretroviral sand others sleep in prayer camps. This causes them to default most often. However as disease progression among these groups becomes rapid they return to the health facility to continue treatment with ARVs. This stresses on the need for increased health education with emphasis on the etiology of the disease both at the health facility level and the community level. Patients’ understanding of the origination and disease outcome to a large extent influences their health seeking behavior.

The study might however suffer some limitations. First, the knowledge index might not include all variables or questions necessary to test the knowledge of ART and PMTCT. This could affect our classification of adequate and inadequate knowledge among respondents. Secondly, the questions we asked about knowledge were all closed ended. It is important to differentiate between knowledge by recall, which implies active knowledge, and recognition of correct answers given to closed options, which implies passive knowledge. Thus, the level of adequate knowledge might be false. However, we adopted open-ended questions in the qualitative interviews, and these findings largely supported the quantitative findings. The study also included HIV positive women who were already on ARVs and had prior adherence counseling and this might influence the knowledge level of respondents. Thirdly, the study did not collect information on other factors that might influence knowledge level and adherence to ART such as facility related factors, stigma and other community related factors. The study did not also observe ART counseling at any of the facilities to see how this is conducted and the kind of information given to clients. Finally, the data from the qualitative study was transcribed in twi before being translated into English. The authors analyzed qualitative data from transcripts that had been translated into English and this might have diluted some richness of the data.

## Conclusions

The knowledge level of HIV positive women on ART and PMTCT are important factors in adherence to ART. High knowledge is associated with access, use and adherence to ART. Although knowledge level on the mode of transmission of HIV/AIDS was high, some of the women also perceived it is transmitted through spiritual means. This indicates that although educational interventions are being implemented, they had not effectively demystified perceptions about the origination and causes of HIV/AIDS. The knowledge level of the mothers influenced their adherence to ART. Improving efforts to better the understanding of HIV positive women on HIV/AIDS and ART will therefore impact positively on their adherence to ART. Further study to explore the knowledge and adherence among both males and females would also be needed to inform policy.

## Competing interests

The authors declare that they have no competing interests.

## Authors’ contribution

The study was conceived and designed by all authors. DB and GDK jointly collected the data under supervision by PAB. DB and PAB were involved in the data analysis and interpretation of the study findings. DB wrote the first draft of the manuscript. All authors reviewed and critically revised the manuscript for important intellectual content and agreed to submit the manuscript for publication. All authors read and approved the final manuscript.

## Pre-publication history

The pre-publication history for this paper can be accessed here:

http://www.biomedcentral.com/1472-6874/13/2/prepub
